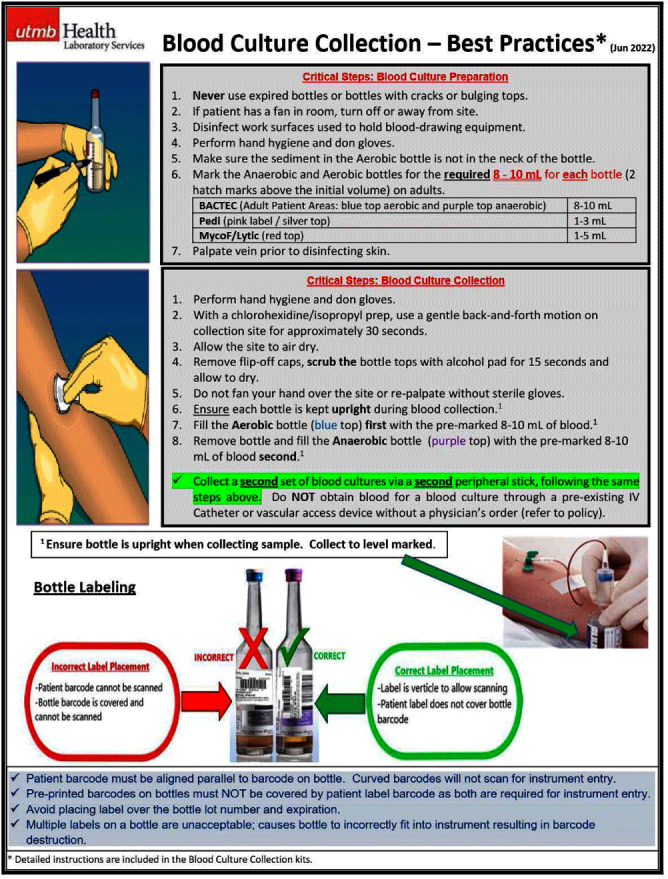# Back to Basics: Blood Culture Contamination Reduction Across a Multicenter Academic Health System

**DOI:** 10.1017/ash.2024.308

**Published:** 2024-09-16

**Authors:** April McDougal, Mary Ann DeMaet, Kassandra Larson, Jean Houk

**Affiliations:** University of Texas Medical Branch

## Abstract

**Background:** Blood culture contamination is common in healthcare and contributes to diagnostic uncertainty, unnecessary treatments and follow-up testing, increased length of stay, higher rates of reportable healthcare-associated infections and events, over utilization of resources and staff including consultative care, and undue emotional stress to patients. The national benchmark for institutional blood culture contamination rates as recommended by The American Society for Microbiology (ASM) and the Clinical Laboratory Standards Institute (CLSI) is < 3 %. Our institution’s overall rate was 8.9% with the highest burden being from our Emergency Department (ED) locations. We formed a multidisciplinary team aimed to reduce these rates through efforts centered around education and simplification of the collection process. **Method:** Working closely with and gathering feedback from nursing staff, nurse educators, and supply chain, we developed a blood culture collection kit that included all items necessary for blood culture collection thus eliminating the need for nurses to gather these items individually prior to collection. Additionally, simple step-by-step educational materials (Fig. 1) detailing the collection technique were provided. Education was presented at nursing huddles and skills fairs prior to kit roll-out. Blood culture kits were then stocked in place of individual blood culture collection bottles in all ED stock rooms in August 2022. **Result:** The 12-week pre-intervention period found 249 contamination events from 4265 total collections deriving a contamination rate of 5.8% across our health system’s four ED locations. During a 12-week post-intervention period following kit roll-out, 116 contamination events occurred from 3629 total collections deriving a contamination rate of 3.2% across our four ED locations. Given our results, we ultimately rolled this out to all units in all locations of our health system. When including all time from kit rollout to present (August 2022 to November 2023, 16 months), there were 1077 contamination events from 43379 total collections deriving an overall contamination rate of 2.5%. When compared to the 16 months prior to the kit rollout (April 2021 to July 2022) there were 1803 contamination events in 49335 total collections (3.7% contamination rate) deriving an overall percent reduction of 32.1%. **Conclusion:** We were able to decrease our health system’s blood culture contamination rate through simple interventions aimed at reducing the mental burden on nursing staff by developing a blood culture collection kit and educational materials. Since implementation of the kits, we have continued to maintain lower contamination rates as evident by our 16 month follow up period.

**Figure:**